# Primum non Nocere: How to ensure continuity of care and prevent cancer patients from being overlooked during the COVID‐ 19 pandemic

**DOI:** 10.1002/cam4.4986

**Published:** 2022-06-27

**Authors:** Veronica Agostinelli, Chiara De Filippis, Mariangela Torniai, Marco Bruno Luigi Rocchi, Alessandra Pagliacci, Giulia Ricci, Rosina Corsi, Paolo Luzi, Michele Caporossi, Rossana Berardi

**Affiliations:** ^1^ Department of Medical Oncology Università Politecnica delle Marche, AOU Ospedali Riuniti Ancona Ancona Italy; ^2^ Department of Medical Oncology Fermo Italy; ^3^ Department of Biomolecular Sciences‐Service of Biostatistics University of Urbino Carlo Bo Urbino Italy; ^4^ TOPS Healthcare Communication srl Rome Italy; ^5^ General Direction AOU Ospedali Riuniti of Ancona Ancona Italy

**Keywords:** cancer management, medical oncology, psychosocial studies, viral infection

## Abstract

**Background:**

Coronavirus disease 2019 (COVID‐19) has spread to all countries since December 2019, triggering a pandemic within weeks of the initial outbreak. Doctors were presented with the challenge of having to reimagine the traditional hospital organisation in order to effectively manage patients.

**Patients and Methods:**

During the months of the COVID‐19 pandemic our Institution was assisted by a call‐center (CC) that triaged cancer patients planned for follow‐up in our outpatient clinics: C1 (for female cancers), C2 (for gastrointestinal, urogenital, and thoracic tumours), and D1 (for melanoma and for patients with tumours in over 5 years follow up). Data refers to the period between 15 April and 3 July 2020.

**Results:**

A total of 1054 patients have been included in our study and 1005 (95%) of the contacts were successful. The analysis showed a majority of female patients (74%) and patients affected by breast cancer (56%). Among the options provided 646 patients (92.4%) opted for online consultancy.

**Conclusion:**

This study has shown that cancer patients valued technology‐mediated follow‐up visits mainly during the beginning of the pandemic because patients themselves were afraid to come to the hospital. Although telemedicine has intrinsic limitations, it is important for providing assistance and preventing cancer patients from feeling isolated during an emergency.

## Graphical abstract

Coronavirus disease 2019 (COVID‐19) has spread to every Country causing pandemic in few weeks. Physicians were asked to redefine ordinary Hospital organization to manage patients. A total of 1054 patients have been included in our study and 1005 (95%) of the contacts were successful. This study shows that cancer patients do appreciate technology‐mediated follow up visit mostly during the beginning of pandemic because patients themselves were afraid to come to the hospital.

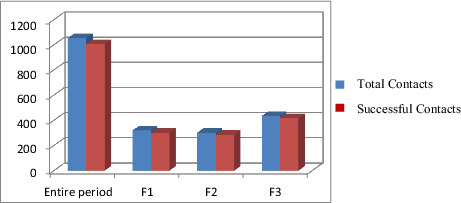

## 1 INTRODUCTION

At present, there are 252,902,685 confirmed cases worldwide of COVID‐19 since the beginning of the pandemic and 5,094,826 deaths.^1^ Furthermore, Italy was deeply affected by the COVID‐19 emergency with the largest outbreaks primarily in the northern regions, but the disease quickly spread throughout the country. Since 9 March 2020, several decree laws have been activated by the Italian Government to limit viral transmission and contagion, constituting emergency protocols, and imposing restrictions that gradually became more severe.^2^ Despite this, 20 months after the beginning of the pandemic, in Italy there were, 860,061 confirmed cases and 132,775 deaths.^1^, ^2^, ^3^The COVID‐19 pandemic placed the Italian National Health System under extraordinary pressure and the demands placed upon it by the pandemic called for a profound reorganisation: Resources were concentrated on the treatment of COVID‐19 affected patients and the majority of deferred activities were suspended.^4^

As a result, ensuring the prompt and appropriate care of all patients suffering from different diseases has become increasingly difficult during this emergency.^5^The oncologic community made great efforts to ensure optimal assistance to cancer patients, despite the need to reduce as much as possible the number of required hospital visits^6^ with the aim of protecting them from contracting COVID‐19.

Due to several potentially concomitant factors such as age, the number of comorbidities, poor performance status, immunosuppressive effects related to the tumour itself and/or systemic anticancer treatments, the cancer patients are at an increased risk of developing severe forms of SARS‐Cov‐2 infection.^7^

However, it is well known that cancer patients should be carefully monitored by clinicians to enable symptoms to be treated as quickly as possible,^8^ so as to ensure an optimal quality of life and an accurate follow‐up.^9^ According to this, oncologists tried to identify new technological ways to ensure continuing care for their patients^10^ effectively introducing the use of telemedicine defined by the WHO and the concomitant American Telemedicine Association as providing virtual clinical services from a distance by using information technology and electronic communications.^11^

During the COVID‐19 era there has been a high implementation of the telemedicine service in cancer care by converting hospital visits into phone consultations preserving the risk‐benefit ratio. What is more, The European Society for Medical Oncology (ESMO) encouraged the use of telemedicine to monitor stable patients and non‐critical patients.^12^ The aim of our study is to assess the role of telemedicine to ensure the optimal ongoing care of cancer patients and to fortify doctor‐patient relationships in such a delicate and particular moment.

## 2 PATIENTS AND METHODS

### 2.1 Study population

During the COVID‐19 pandemic, our work was assisted by a call‐center (CC, named TOPS s.r.l.) to triage cancer patients programmed for follow‐up appointments in our outpatient clinics.

In this retrospective study, data collected refers to the period from 15 April 2020 to 3 June 2020. The CC tried to call a total of 1054 patients from 7 April 2020 to 24 June 2020, of which 1005 patients were contacted successfully.

### 2.2 Inclusion and exclusion criteria

The inclusion criteria were:

- age ≥ 18 years old;
- male or female;
- any cancer diagnosis;
- patients in follow‐up;
- patients with newly diagnosed cancer.

The exclusion criteria were:

- patients undergoing active treatment (excluding hormone therapy);
- patients who require active supportive therapy.

### 2.3 Data collection

The CC activity was divided into three phases. During the first phase (F1), from 15 April 2020 to 8 May 2020, CC contacted 319 patients to cancel their appointments which had been postponed by their oncologist. In the second phase (F2), from 11 May 2020 to 5 June 2020, 301 cancer patients and in the third phase (F3), from 11 June 2020 and 3 July 2020, 434 patients were contacted, respectively.

In the second and third phases, CC asked patients their preferred way to relate with the physician. Patients could select between different options: If they preferred not to go to the hospital, they could choose between a phone call visit or a web‐video call placed through a dedicated platform (Google Meet), or alternatively, it was possible to use WhatsApp. Patients were also able to decide to come to the hospital for a physical examination; this option was kept and not postponed in case of clinical needs and initial consultations.

All the patients were divided into three different outpatient clinics: C1 specialised in breast and gynaecological diseases, C2 was dedicated to patients with gastrointestinal, urogenital, and thoracic tumours, and D1 dealt with patients affected by melanoma and patients with any type of tumour in over 5 years follow up.

Furthermore, for each patient we also considered clinical‐pathological data including: Sex, age, outpatient clinic, type of pathology, stage of pathology, active therapy and which type of therapy was being used in their treatment, in order to correlate all these characteristics with the preferred visit type.

### 2.4 Statistical analysis

In our study, we tried to evaluate if the visit modality chosen by patients could be related to some of their characteristics. The following variables were considered: phase (2 vs. 3), sex (male vs. female), age (<50, 50–75, >75 years old), type of cancer (breast, gastrointestinal, prostate, genitourinary, lung and chest, melanoma, gynaecological, other type), therapy (active vs. non active).

To identify the variables influencing the choice of visit modality, a multimodal regression model was applied and within each potential predictive variable, a category has been fixed as a reference category.

All the statistical analyses were carried out at a significance level of 0.05. The analyses were performed using SPSS and R software.

## 3 RESULTS

**FIGURE 1:**
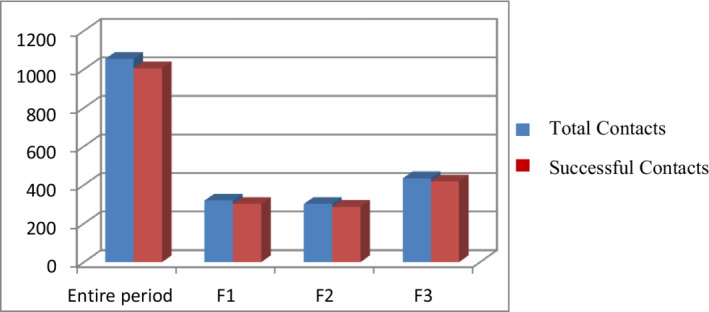
Successful contacts for the entire period and for each phase

In our study, 1054 patients were enrolled retrospectively: among these, 1005 patients (95%) were successfully contacted by CC (Figure 1) and a gender‐stratified analysis showed a majority of female patients (780; 74%). We also divided patients by age: Hundred and seven patients (10%) were less than 50 years old, most patients, 623 (59%), were between 50 and 75 years old and 324 patients (31%) were above 75 years old.

**FIGURE 2:**
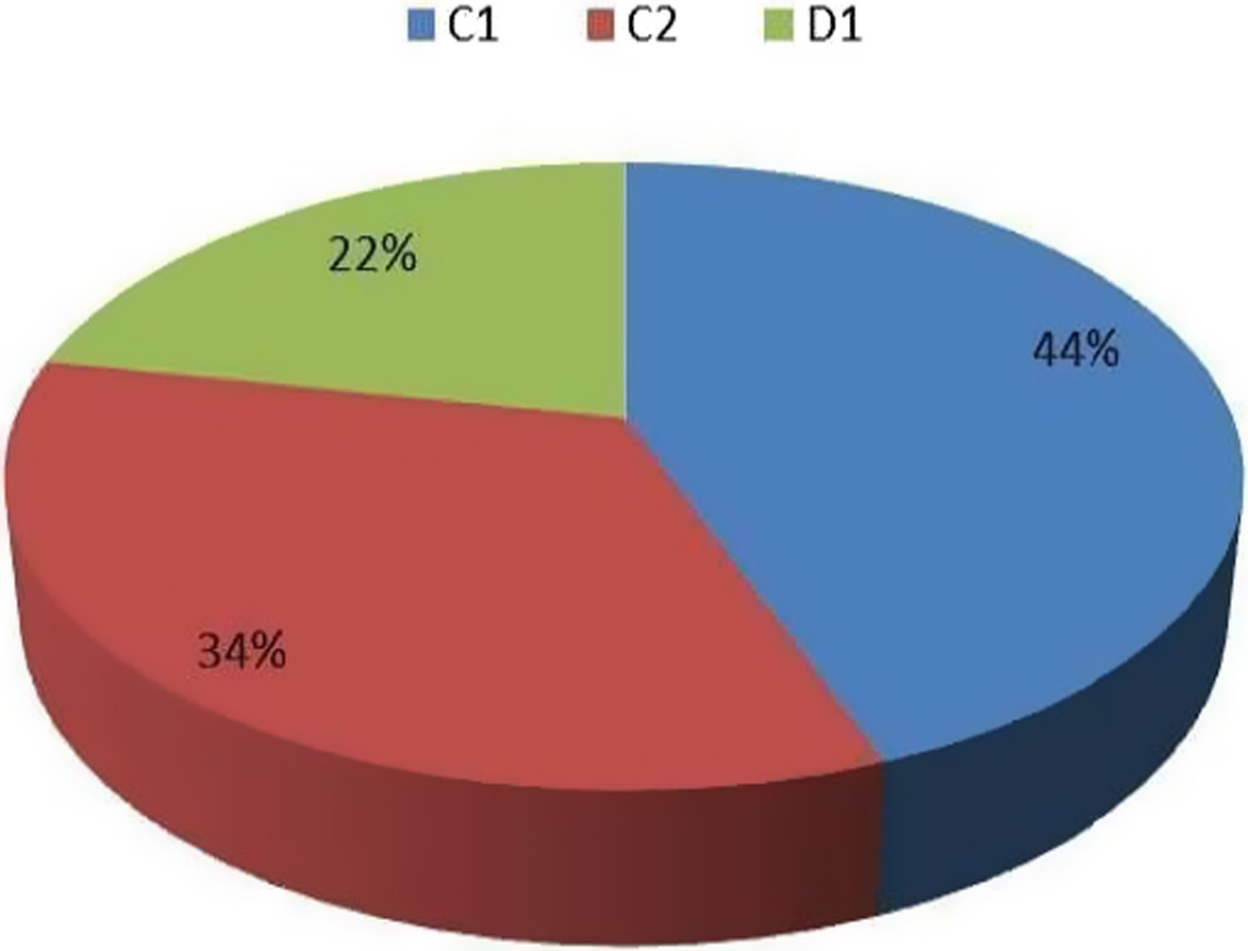
Outpatients of all patients

The patients were then allocated between the three different outpatient clinics: 467 patients (44%) in C1, 356 patients (34%) in C2 and 231 ones (22%) in D1 (Figure 2).

**FIGURE 3:**
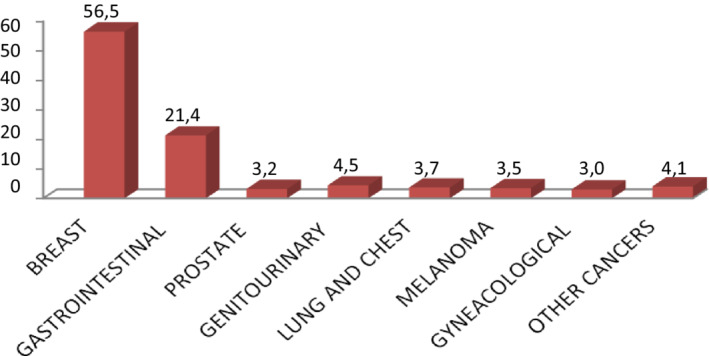
Types of pathology of all patients

According to these data, among all the patients enrolled in the study, breast cancer was the most frequent disease (596 pts, 56%), followed by gastrointestinal (GI) cancer in 226 patients (21%). In Figure 3 and Table 1 we summarised all the diseases.

**TABLE 1:** Total patients characteristics

Characteristics	Value (%)
Total	1054 (100)
Phases	
F1	319 (30)
F2	301 (29)
F3	434 (41)
Patients contacted	1005 (95)
Patients uncontacted	49 (5)
Outpatient	
C1	467 (44)
C2	356 (34)
D1	231 (22)
Male	274 (26)
Female	780 (74)
Age	
< 50	107 (10)
50–75	623 (59)
> 75	324 (31)
Type of pathology	
Breast cancers	596 (56)
Gastrointestinal cancers	226 (21)
Prostate cancers	34 (3)
Genitourinary cancers	47 (5)
Lung and chest tumours	39 (4)
Melanoma	37 (4)
Gynaecological cancers	32 (3)
Others cancers	43 (4)
Staging	
Low	730 (69)
High	192 (18)
Unknow	132 (13)
Therapy	
No	602 (57)
Yes	452 (43)
Type of therapy	
Aromatase inhibitor (AI)	323 (31)
AI + aLH‐RH	30 (3)
Tamoxifen	65 (6)
Tamoxifen + aLH‐RH	18 (2)
Androgen deprivation	16 (1)

Moreover 452 patients (43%) in our study were undergoing anticancer therapy as shown in Table 1.

Furthermore, taking into consideration the spread of the pandemic, we considered it to be appropriate to divide all the patients into three groups according to three time phases. F1 included 319 patients (30%) with a scheduled outpatient visit from April 15th to May 8th, F2 301 patients (29%) scheduled from May 11th to June 5th, F3 434 patients (41%) scheduled from June 11th to July 3rd.

During F1, all patients were called by CC to cancel appointments that were subsequently rescheduled by our physicians: in that period the hospital was not a safe place and often the patients themselves were afraid to go there.

**FIGURE 4:**
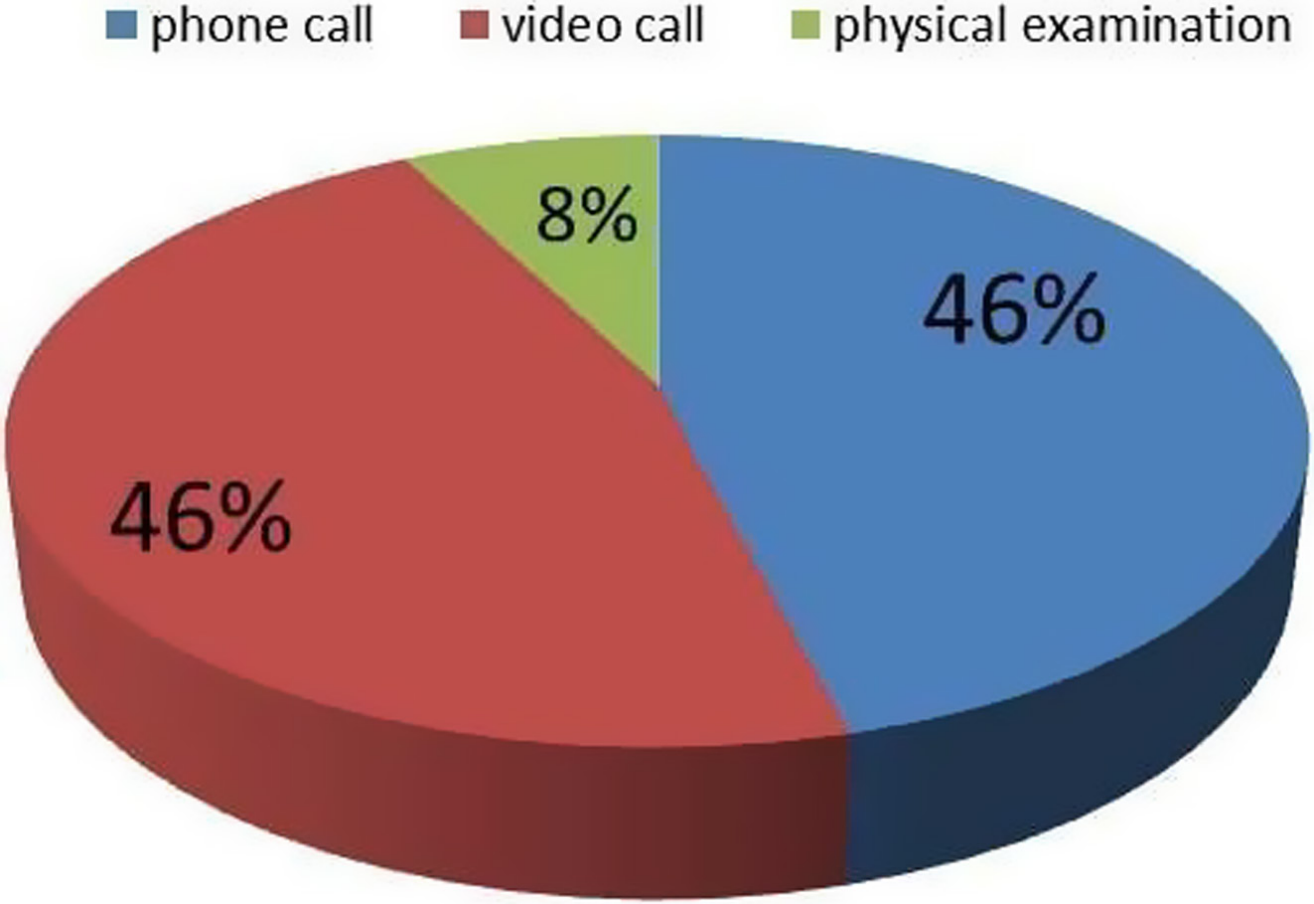
Types of visit preferred by patients

Instead, during F2 and F3 CC asked the 698 contacted patients which type of visit they preferred. Most of them chose a remote consultation: 324 (46,4%) opted for a video call using WhatsApp or a dedicated platform (Google Meet) and 322 patients (46%) selected a phone consultation; only 53 patients (7,6%) expressed their intention to come to the hospital for examination (Figure 4).

The multivariate analyses of F2 and F3, considering sex, age, type of pathology and use of active therapy, showed that remote consultations were significantly preferred during F2 (type of visit by phone vs. in person: 4.16, CI 95% 1.86–9.29, *p* = 0.001; by web call vs. in person: 5.28, CI 95% 2.37–11.74, *p* < 0.001). It is worth noting that F2 was shortly after the beginning of the pandemic, whereas during F3 the spread of disease and hospitalisation due to COVID‐19 was decreasing.

Furthermore, male patients seemed to prefer a physical examination compared to a remote consultation (type of visit by phone vs. in person: 0.25, CI 95% 0.09–0.69, *p* = 0.007; by web call vs. in person: 0.34, CI 95% 0.12–0.94, *p* = 0.038)

Lastly, the choice of a remote consultation was found to be significantly preferred among patients with lung or chest tumours (type of visit by phone vs in person: 136.96, CI 95% 115.02–151.91, *p* < 0.0001; by web call vs in person: 127.89, CI 95% 112.53–153.12, *p* < 0.0001). There was no significant relationship discovered between age or use of active therapy. The results of multivariate analysis are shown in detail in Table 2.

**TABLE 2:** Multivariable analyses for type of visit modality

Test variables	Phone call OR (95% CI)	*p* value^a^	Web call OR (95% CI)	*p* value^a^
F2	4.16 (1.18–9.29)	0.001	5.28 (2.37–11.74)	< 0.0001
F3^b^				
Male	0.25 (0.09–0.69)	0.007	0.34 (0.12–0.94)	0.038
Female^b^				
Age < 50 years	0.46 (0.14–1.51)	0.2	2.03 (0.65–6.41)	0.23
Age 50–75 years	0.63 (0.31–1.25)	0.18	1.19 (0.59–2.4)	0.64
Age > 75 years^b^				
Breast cancers	0.70 (0.13–3.66)	0.67	1.35 (0.25–7.23)	0.73
Gastrointestinal cancers	1.88 (0.42–8.44)	0.41	2.8 (0.61–12.87)	0.19
Prostate cancers	0.97 (0.14–6.68)	0.97	1.8 (0.36–12.5)	0.55
Genitourinary cancers	1.35 (0.21–8.62)	0.75	2.13 (0.34–13.48)	0.42
Lung and chest tumours	136.96 (115.02–151.91)	< 0.0001	127.89 (112.53–153.12)	< 0.0001
Melanoma	4.07 (0.35–47.2)	0.26	4.89 (0.41–57.7)	0.21
Gynaecological cancers	1.20 (0.09–16)	0.89	1.55 (0.11–21.55)	0.75
Others cancers^b^				
Active therapy	1.27 (0.56–2.85)	0.57	1.21 (0.54–2.73)	0.64
No active therapy^b^				

^a^ Statistically significant (*p* < 0.05).

^b^ Reference categories.

The multivariate analyses of separate F2 and F3 seemed to confirm the previous data. During F2, patients allocated to outpatient clinic C1 preferred a remote consultation compared to coming to the hospital (type of visit by phone vs in person: 10.48, CI 95% 1.73–63.59, *p* = 0.01; by web call vs. in person: 5.79, CI 95% 1.07–31.12, *p* = 0.042). patients of C2, instead, seemed to significantly prefer physical examination (type of visit by phone vs. in person: 9.34E‐07, CI 95% 1.82E‐08–4.8E‐05, *p* < 0.0001; by web call vs. in person: 5.95E‐07, CI 95% 1.31E‐0.8–2.69E‐05, *p* < 0.0001).

Nevertheless, patients with breast cancer preferred to come to the hospital for their visit compared to receiving a phone consultation (type of visit by phone vs in person: 8.09E‐08, CI 95% 1.5E‐08–4.36E‐07, *p* < 0.0001).

Conversely, the analyses showed that, during F3, patients preferred to come to the hospital rather than have a remote consultation. Only patients with a lung or chest tumour seemed to prefer a phone consultation (type of visit by phone vs in person: 149.31, CI 95% 89.12–190.57, *p* < 0.0001).

## 4 DISCUSSION

“Primum non Nocere” (First, do no harm, attributed to Hippocrates) is one of the core ethical principles that physicians adhere to.^13^ Over the past months, the COVID‐19 pandemic has placed the health care system under immense pressure and has forced a complete reorganisation.

The most significant contradiction is related to the virus's introduction into hospitals, which are considered to be the very heart of health care, the place where citizens are treated and where cancer patients are protected: the patients themselves were afraid of going to the hospital.^14^

At the beginning of the emergency, physicians felt the need to protect patients, especially the weakest, postponing appointments, when feasible, to avoid putting patients at risk. As the pandemic progressed, it became ever more necessary to find an alternative solution to traditional in‐person consultations. Owing to this, the American Medical Association has promoted the use of telemedicine to continue patient care during the COVID‐19 pandemic.^15^

Also in Italy, to underline the importance and the ever increasing use of telemedicine as a way of consulting the patient, in December 2020, the state‐regions conference signed an agreement to ensure that telemedicine would become part of the National Health System (SSN) as an essential assistance service.^16^, ^17^ Telemedicine has emerged as a safe and practical option for increasing access to patient care, lowering the risk of Sars‐CoV‐2 infection in both patients and health care providers, and reducing demand for personal protective equipment, which was scarce during the early phase of the pandemic.^18^ In fact, to reduce the need for hospital visits, National Health Service (NHS) England guidelines also recommend the use of telephone or video consultation in the care of cancer patients who do not have COVID‐19.^19^

Telemedicine was first implemented some years ago, but with the pandemic its use has become more prevalent.^20^ Through this tool, it was possible to communicate and retain patient health information by contacting patients at home. Telemedicine and virtual software platforms provide an inexpensive, effective, and appealing solution in this setting.^21^

Outpatient visits were one of the first settings in which telemedicine was implemented, thus maintaining the doctor‐patient relationship without increasing the risk of infections, as well as minimising needless interaction in waiting rooms and during transportation to the hospital.^22^

It has been shown that the use of telemedicine results in a high level of user satisfaction, especially when it simplifies consultations for many patients who must travel long distances to have a medical examination by an expert.^23^

Among cancer patients, telemedicine was even more useful because of the particularly negative impact the pandemic has had on these patients, who have been cut off from a formal and familiar health care environment that is linked with their cancer trajectory.^24^

Although the use of telemedicine holds promise for managing pandemic response, it is important to highlight that it also presents challenges and limitations such as the availability of robust infrastructure, equipment costs, as well as physician and nurse training.^25^ Ethical aspects, among others, must also be taken into account.^26^

Telemedicine necessitates a technologically savvy population with prior exposure to internet‐based technology. It is worth considering that a significant number of cancer patients are of an older generation or live in rural places, so the use of telemedicine becomes an impediment owing to limited access to telemedicine platforms and poor internet access.^27^

Additionally, patients may have started to experience anxiety and nervousness due to the use of technology and isolation.^28^ In fact, during the pandemic, social distancing and quarantine have been adopted as efficient strategies of reducing COVID‐19 diffusion.^29^

Furthermore, medical practice is largely compromised by the limitation of physical examination.^30^ In‐person visits provide patients with greater privacy, allow them to better comprehend if something is wrong, satisfy their needs for supportive treatment, and allow them to establish a trusting relationship.^31^

Our study underlined that the use of telemedicine has been highly appreciated by patients, with more than 90% of the enrolled patients preferring to carry out a telematic consultation rather than come in person to the hospital.

This situation was most prevalent in the early stages of the pandemic: our study showed that telematic visits were significantly preferred during F2 compared to F3.

Conversely, the more the spread and hospitalisation of COVID‐19 patients decreased, the more cancer patients preferred to come back to the hospital for their physical examination.

Despite an approved platform for telemedicine, through Google Meet, being available at our centre, the majority of patients chose a video call with WhatsApp as their preferred method of consultation, highlighting how important it is to use simple tools among cancer patients: they are often elderly and without the support of younger family members, especially during the height of the pandemic. It is worth noting that 31% of the patients included in this study were over 75 years old and 90% were over 50 years old.

WhatsApp is a widely used tool for communication because it helps people to stay connected. Its great popularity can be attributed to the fact that it only requires the use of a smartphone and mobile internet connection and this simplicity also makes it accessible for older patients.^32^ Despite the fact that scientific studies on the use of WhatsApp are limited in the medical literature, a rising number of health professionals have used it as a communication platform.^33^

Gender also seemed to influence the choice of visit modality: Analysing F2 and F3 together, the results showed that males preferred an in‐person visit. Moreover, taking into consideration only F2, it was seen that the patients scheduled in the C1 clinic (mammary and gynaecological tumours), therefore mainly women, preferred an electronic visit modality, even if the patients with breast cancer, on the contrary, seemed to prefer hospital access.

Patients with lung or chest cancer consistently preferred telematic visits both when considering F2 and F3 together but also considering only F3, when in general patients slowly began to want to return to in‐person visits. Most likely, in view of the nature and dangers presented by COVID‐19 infection, lung cancer patients were much more frightened of and worried about a possible infection.

Our study has some limitations. Firstly, it considers a very limited period of time and for this reason the study consists of a relatively small number of patients. Moreover, it refers to the first phase of the pandemic, when we were completely unaware of its evolution and the consequences it may have had from various perspectives.

Secondly, it would have been interesting to know the patients' residence data so as to understand if the distance from the hospital might have influenced the choice of consultation type. Our centre is a point of reference in our region, so that it is frequently visited by patients who live far away. Probably, in a period in which travel was restricted, this aspect may have affected patients' choice of consultation.

Moreover, it would have been interesting to expand upon our data with those from other centres.

Despite all the limitations, for our centre, our data have been important to corroborate and expand the use of telemedicine during the pandemic.

Telemedicine may be utilised to give proactive and continuous encouragement to cancer patients in the future combining this mode with traditional in‐person visits. Certainly, face‐to‐face interaction between physician and patient remains the gold standard of clinical treatment, but it may be appropriate to reserve telemedicine for emergency situations, remote monitoring, and inter‐professional consultations.

## 5 CONCLUSION

The pandemic has been a significant challenge for the healthcare system, as virtual health skills, such as doing physical examinations without contact and without losing empathy for the patient or affecting the quality of their care, have had to swiftly develop.

Even if physical examination is one of the most important moments in the relationship between patient and physician, it is also important to ensure continuity of care during an emergency and telemedicine should be considered the best way to guarantee this.

Moreover, in the future, it could be interesting to further integrate physical examinations with telematic consultations, ensuring in this way, a better and more complete healthcare approach for all patients.

## AUTHOR CONTRIBUTIONS

Conception and design: Rossana Berardi, Veronica Agostinelli, Mariangela Torniai. Administrative support: Michele Caporossi. Provision of study materials or patients: Veronica Agostinelli, Mariangela Torniai, Alessandra Pagliacci, Giulia Ricci, Paolo Luzi, Rosina Corsi. Collection and assembly of data: Veronica Agostinelli, Chiara De Filippis. Data analysis and interpretation: Marco Bruno Luigi Rocchi. Manuscript writing: Veronica Agostinelli, Chiara De Filippis, Mariangela Torniai, Rossana Berardi. Final approval of manuscript: Veronica Agostinelli, Chiara De Filippis, Mariangela Torniai, Rossana Berardi, Marco Bruno Luigi Rocchi.

## CONFLICT OF INTEREST

The authors declare no conflict of interest with the present study.

## ETHICAL APPROVAL STATEMENT

The study was conducted in accordance with the precepts of Good Clinical Practice and the ethical principles of the Declaration of Helsinki. Results presented in this article contain no personally identifiable information from the study.

## INSTITUTIONAL REVIEW BOARD STATEMENT

According to Italian law (resolution 1 March 2012, Gazzetta Ufficiale n.72 of 26 March 2012), ethics approval was not required for the present study.

## INFORMED CONSENT STATEMENT

Informed consent was not required because of the retrospective nature of the study.

## Data Availability

Data sharing is not applicable to this article as no new data were created or analyzed in this study.
